# Myasthenia Gravis: utilising cross-platform quantitative content analysis to uncover and validate unmet needs

**DOI:** 10.3389/fneur.2024.1474347

**Published:** 2024-09-27

**Authors:** David Legg, Andreas Meisel, Maike Stein, Lea Gerischer, Meret Herdick, Daniela Krüger, Philipp Mergenthaler, Lars Masanneck, Sophie Lehnerer

**Affiliations:** ^1^Department of Neurology with Experimental Neurology, Charité—Universitätsmedizin Berlin, corporate member of Freie Universität Berlin and Humboldt-Universität zu Berlin, Berlin, Germany; ^2^Centre for Stroke Research Berlin, Charité—Universitätsmedizin Berlin, Berlin, Germany; ^3^Health Services Research in Emergency and Acute Medicine, Charité—Universitätsmedizin Berlin, Berlin, Germany; ^4^Neuroscience Clinical Research Centre, Charité—Universitätsmedizin Berlin, corporate member of Freie Universität Berlin and Humboldt-Universität zu Berlin, Berlin, Germany; ^5^Berlin Institute of Health at Charité—Universitätsmedizin Berlin, Digital Health Centre, Berlin, Germany; ^6^Radcliffe Department of Medicine, University of Oxford, Oxford, United Kingdom; ^7^Department of Neurology, Medical Faculty and University Hospital Düsseldorf, Heinrich-Heine-University Düsseldorf, Düsseldorf, Germany

**Keywords:** Myasthenia Gravis, peer-to-peer support, social media, online health communities, online forums, content analysis

## Abstract

**Background:**

Recent years have seen a rapid growth in the number of online health communities targeted at patients with long-term conditions. Myasthenia Gravis (MG) is a rare neurological disease for which such communities have not been analysed before. The aim of this study was to better understand the needs of the MG population through the collation and categorisation of questions that users of MG social media were asking fellow users on these platforms.

**Methodology:**

Systematic observation of four MG Facebook groups was conducted over a 2-month period. Groups were selected for analysis based on the following systematic criteria: Language (English), Membership (≥ 5,000 members), group activity (≥ 2 posts per week), target audience (general MG population) and researcher engagement with group administrators. The study protocol was reviewed by the institutional review board of the Charité—Universitätsmedizin Berlin (EA2/106/22). During the observation period, data were extracted from individual posts featuring questions made across each group using a systematic and objective coding scheme. All data points were coded directly from the source and collated into an SPSS database (IBM SPSS V.27, SPSS). Absolute and relative frequencies were calculated for categorical variables and proportions were compared across groups to validate the credibility and relevance of different requests.

**Results:**

Of the 2,062 posts observed (*N* = 2,062), 1,392 featured questions (*n* = 1,392). Questions were asked by 787 unique users: 531 were identified as one-time users (67%) and 256 were identified as repeat users (33%). Six hundred and fifty six users were classified as presumed diagnosed (83%), 61 as seeking diagnosis (8%), 69 as family and/or friends (9%) and as other (<0%). Eight unique categories of questions were observed including MG treatment (31%), Symptoms (19%), Living with MG (12%), Diagnosis (10%), non-MG medication (11%), Tests (8%), Location (4%) and Other (4%).

**Conclusion:**

Members of the MG population make active use of online health communities to seek and discuss practical information concerning various aspects of the disease, its diagnosis and care. The openness and willingness of the sample population to share sensitive medical information shows a high need for information not entirely catered to by the medical profession.

## Introduction

Myasthenia Gravis (MG) is an antibody-mediated chronic disease affecting the neuromuscular junction causing severe fluctuating muscles weakness (1). Classified as a “rare” disease owing to the fact that it affects approximately one in every 5,000 within the general population (2), cases of MG tend to follow a bimodal distribution with a first peak around 30 years of age, predominantly affecting women, and a second peak after 50 years of age, with a higher prevalence of men (3). Diagnosis of the disease is complicated by the clinical features and antibodies involved, most notably in cases of double seronegative MG in which the main antibodies used to detect the disease tested are not present (4).

Aetiology aside, owing to their broad geographical distribution, the affected population are faced with a unique set of challenges (5–7). Foremost amongst these challenges is access to appropriate care and peer-to-peer support: an integral aspect of self-management for individuals with chronic illnesses (8). Regarding the latter, individual’s social networks have been shown to play an important role in managing psychological challenges as well as maintaining overall well-being (9). Whilst friends and family are integral to these support networks, individuals have been shown to place a high value on communicating with peers who have had a similar experience (10). Accordingly, recent years have seen an increase in the number of online health forums and discussion boards as patients and caregivers search for information and seek peer-to-peer support online (11).

Rare diseases such as MG are no different in this regard. Evidence suggests online health communities are considered particularly valuable by affected patient populations and their caregivers and that the use of and participation in such groups has become a regular activity for many (12, 13). Resultantly, users of these online platforms have generated a great volume of data, which has the potential to advance understanding of patient populations and improve patient care (14–16). With that said, though much has been made of the promise of social media to improve understanding of rare diseases (17–20), few published studies have taken advantage of the depth of information generated by the users of online platforms. In point of fact, in their systematic review of social media research in rare genetic diseases, Miller et al. (21) found that a majority of studies were surveys that did not make use of the large volumes of data generated by users of these platforms.

Whilst it is important to recognise the limitations of this type of self-reported data, namely, difficulties in verifying the authenticity of users’ medical conditions (22), these online resources may offer invaluable insight into hard-to-reach patient populations. Bearing this in mind, this study has two primary objectives. First, to collate and categorise the questions users of MG social media and online forums are asking fellow users to better understand the needs of the MG population. Second, to compare the frequency of question categories posted across different social media platforms and online forums in order to validate the authenticity of different request. If the same type of question is observed across multiple independent groups, interest in the topic amongst the sample population can be attributed a higher level of credibility and relevance.

## Methodology

### Study design

Systematic observation of four popular MG Facebook groups was conducted over a 2-month period (22.03.2023–22.05.2023). During the observation period, data were extracted from individual posts featuring questions made across each of the participating groups. Only the content of the posts themselves were analysed. Questions were defined as requests for information and were identified through the use of standard question words such as How, Who, What, When, or Why and punctuation indicative of a question (“?”). All other posts were excluded from the analysis.

Data were extracted using a systematic and objective coding scheme created following a month-long immersion process in which the primary research noted the different type of questions made by group members and recorded the number of posts made per day across each of the participating groups (01.01.2023–31.01.2023). The coding scheme was then ratified by two practising neurologists (SL and MS) and tested on a further publicly available MG health forum with the assistance of a third independent researcher (DK). The final coding scheme can be found in the Supplementary material 1.

### Sample selection

Groups were selected for analysis using a two-stage search strategy in order to identify relevant forums. First a Google and Social Media Platform Search was conducted. The Social Media Platforms searched included Facebook and Reddit. These platforms were selected due to their format and prominence (number of monthly active users and type of content hosted: primarily text). The second stage focused on official Myasthenia Gravis Organisations/Charities Websites. This was carried out in order to identify any forums/groups which had official backing. Relevant Organisations and Charities were identified through membership of European Association of Myasthenia Gravis Patients Associations (EUMGA).

In total, 80 Facebook groups concerning Myasthenia Graves in Humans were identified. Two Facebook groups were identified as concerning Myasthenia Gravis in Dogs. Four other platforms were identified as hosts of myasthenia gravis forums. Groups were excluded and included for analysis based on the following systematic criteria: Language (English), Membership (≥ 5,000 members), group activity (≥ 2 posts per week), target audience (general MG population: no specified sub-group, e.g., country or gender based) and researcher engagement with group administrators. Further details of the search strategy used to create the sampling frame can be found in Supplementary material 2.

### Coding

All data points were coded directly from the source and collated in an SPSS database (IBM SPSS V.27, SPSS). Due to the ephemeral nature of posts made to the platform, coding was conducted on daily basis. All posts were ordered from new to old using the “sort” function to enable researchers to capture all of the latest posts. To ensure the accuracy of the data extraction process the two independent researchers (DL and SL) made regular comparisons to ensure the validity of the coding scheme and quality of the data extraction. Data were only finalized following consensus. Following the observation period, author data points including gender and diagnosis status were cross-referenced to ensure there was no differences between the categorisation of authors who made multiple posts. All discrepancies were resolved by consensus and reference to the extracted data points.

### Variables

Variables of interest included the following items: pseudonymized group code, encrypted profile code, gender (male; female; unknown), author diagnosis status (presumed diagnosed; seeking diagnosis; friend or family; and other), textual location information (if available), number of questions included in post, question category and subcategory. Only information included within the posts were analysed. No individual group user profiles were accessed. Questions featured in posts with multiple questions were treated as individual questions, marked as coming from the same route post, and categorised separately.

One-time and repeat users were differentiated by cross-referencing profile code and route post. One-time users were defined as users who made one post during the observation period and repeat users were defined as users who made more than one post. Importantly, a one-time user who made a post that contained more than one question was still identified as a one-time user.

### Analysis

The focus of the analysis was on the frequency of author and question categories recorded during the observation period. Absolute and relative frequencies (proportions) are presented for categorical information. Categories were considered validated by the cross-platform analysis if they featured posts from two or more distinct users in three or more of the participating groups. Chi2 was used to test for the significance of differences in the proportion of user categories in each group. A one-way ANOVA test was used to establish whether the differences in the proportion of question categories asked by different author categories were statistically significant. All analyses were performed with SPSS.

### Ethics

The study protocol was reviewed by the institutional review board of the Charité—Universitätsmedizin Berlin (EA2/106/22) and conducted in accordance with the standards recommended by the Association of Internet Researchers (23) and GDPR (24). All data were stored on a secure university server. Access was restricted to the core research team and password protected.

In line with recommended practise for studying private “closed” online groups, the researchers identified themselves to and sought the consent of each group’s gatekeeper of the community (e.g., site admin) to conduct the research before making the wider group aware of the objectives. Study information (see Supplementary material 3) was made publicly available to groups via a “pinned post” (25) with a link to further information on the user profile of the primary researcher (DL) which had been created for this purpose. In this way Facebook group members were made aware of the research and were given the chance to opt out prior to the study commencement and throughout the observation period. All data were coded directly from the source.

Groups were pseudonymized and individual posters profile codes were encrypted using the Kutools for Microsoft Excel add on (26). Re-identification of the data was only possible with a randomly generated eight-digit decryption key. Following the completion of the observation period the decryption key and accompanying database were deleted so as to partially anonymise the data. Re-identification of the dataset by a third party could be possible but only with great effort (27). Participation could not be revoked following the partial anonymization of the data.

### Termination criteria

To ensure the researchers’ presence did not have a negative impact on the nature of the group, comparisons were made between the frequency of group members’ posts in the immersion period and first 2 weeks of the formal observation period. It was agreed within the research team, a decrease of ≥30% in the average number of posts made in each group within the first 2 weeks of observation was grounds for study termination.

## Results

Of the 2,062 posts observed (*N* = 2062), 1,392 featured questions (*n* = 1,392). Table 1 displays an overview of the user demographics, author categorisation, and patterns of usage by group. Questions were asked by 787 unique users (*n* = 787). Six hundred and eighty six were female (87%), 97 were male (12%) and four could not be identified due to lack of information (1%). The majority of users were categorised as presumed diagnosis (83%). 8% were classified as seeking diagnosis and 9% were classified as a family or friend. The one user classified as other described herself as a healthcare professional. With the exception of Group 4 in which no Friends or Family were identified, the proportion of different user classifications were similar across all groups. The Chi^2^ test indicated that the proportion of different user categories across all four groups were not significantly different. (*x*^2^ = 3,795, *p* = 0.924).

**Table 1:** Overview of user demographics, author categorisation and patterns of usage.

	*Unique users (n = 787)*	*Group 1 users (n = 607)*	*Group 2 users (n = 121)*	*Group 3 users (n = 86)*	*Group 4 users (n = 22)*	*Multiple group users (n = 37)*
*Gender, n (%)*						
Female	686 (87)	526 (87)	104 (86)	77 (90)	19 (86)	28 (76)
Male	97 (12)	77 (13)	17 (14)	9 (10)	3 (14)	9 (24)
Unknown	4 (0)	4 (0)	0 (0)	0 (0)	0 (0)	0 (0)
*Author category, n (%)*						
Presumed diagnosed	656 (83)	504 (83)	102 (84)	69 (80)	19 (86)	28 (76)
Seeking diagnosis	61 (8)	49 (8)	8 (7)	9 (10)	3 (4)	6 (16)
Friends/Family	69 (9)	53 (9)	11 (9)	8 (9)	0 (0)	3 (8)
Other	1 (0)	1 (0)	0 (0)	0 (0)	0 (0)	0 (0)
*User category, n (%)*						
One-time users	531 (67)	418 (69)	83 (69)	68 (79)	17 (77)	n/a
Repeat users	256 (33)	189 (31)	38 (31)	18 (21)	5 (23)	37 (100)
Mean	Mean – 3	Mean – 3	Mean – 3	Mean – 2	Mean – 2	
Mode	Mode – 2	Mode – 2	Mode – 2	Mode – 2	Mode – 2	

37 repeat users (14%) were observed participating across one or more of the observed groups. The unique user column refers to the total number of users minus repeat users who were observed posting the same type of MG treatment question across multiple groups. The mean depicts the average number of posts made by repeat users, and the mode refers to the most common number of posts made by these users.

Based on the frequency of posts made by unique profile codes, the majority of the sample population were identified as one-time users (*n* = 531, 67%). Two hundred and fifty six identified as repeat users (33%). Repeat users were not restricted to a single group: 37 (14%) of the 256 repeat users were observed participating across multiple groups. The modal number of posts featuring questions made by all repeat users was two across and within each of the four groups. One hundred and twenty two repeat users posted more than twice during the observation period (48%).

Location information was not available for a majority of the sample. Only 96 users (12%) provided location information within the text of their post such as the name of a country, city, or state and/or location specific information such as governmental programmes, e.g., Medicare. Based on this information, 83 users were located in the United States of America (USA) (11%), eight in Europe (1%), three in Canada (<1%), one in India (<1%), and one in South Africa (<1%).

### Question categories

**Figure 1:**
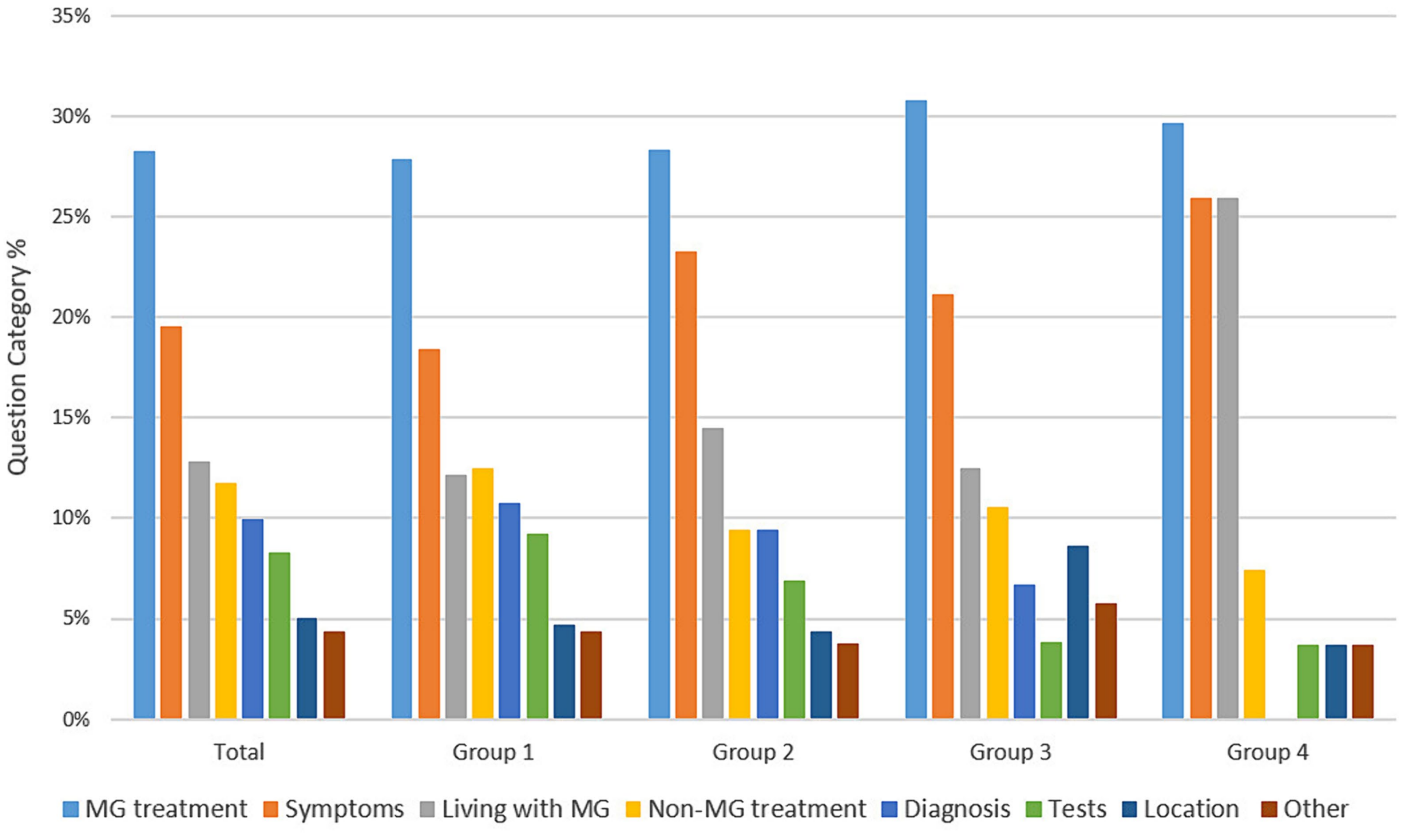
Frequency of users posting different question categories.

Throughout the course of the observation period users of all four participating groups asked questions that could be classified into one of the following eight categories: MG treatment (31%), Symptoms (19%), Living with MG (12%), Diagnosis (10%), Non-MG medication (11%), Tests (8%), Location (4%), and Other (4%). The proliferation of distinct users posting different question categories in each group (Figure 1) indicates that the overall frequency of question categories was not attributable to a small number of prolific users asking about the same topic.

Whilst the frequency of question categories asked by authors categorised as “Presumed Diagnosed” or “Friend/Family” followed a similar pattern to one another (Table 2), the results of one-way ANOVA demonstrated that there was a *statistically significant difference between the categories of questions asked about by different author categories [F (3,1,392) = 9.750, p ≤ 0.001].* This was most evident in the proportion of the sample population asking questions in the Test and Diagnosis subcategories.

**Table 2:** Frequency of question categories asked by different author categories in all four groups.

	*MG treatment*	*Symptom*	*Living with MG*	*Non-MG treatment*	*Diagnosis*	*Tests*	*Location*	*Other*
*Author category, n (%)*								
Presumed diagnosed	371 (33)	203 (18)	152 (14)	129 (12)	104 (9)	63 (6)	49 (4)	48 (4)
Seeking diagnosis	16 (13)	46 (28)	8 (5)	16 (10)	28 (17)	38 (23)	5 (3)	3 (2)
Friends/Family	36 (34)	20 (19)	10 (9)	16 (15)	8 (7)	5 (5)	7 (7)	14 (4)
Other	0 (0)	0 (0)	0 (0)	0 (0)	0 (0)	1 (100)	0 (0)	0 (0)

% refers to the rate of questions categorised into one of the eight categories for each respective author category.

### Question subcategories

Questions concerning MG treatment were the most common type of question across all participating groups. In total, 305 users asked 439 questions related to MG treatment. All questions were further subcategorised to reflect eight distinct categories of MG treatment queried. These included: Symptomatic treatment (*n* = 97; 22%), Biologicals (*n* = 94; 21%), IVIG (immunoglobulins) (*n* = 65; 15%), General (non-specific) (*n* = 48; 11%), Corticosteroids (*n* = 47; 11%), Long-term immunosuppressants (non-corticosteroid) (*n* = 37; 8%), Thymectomy (*n* = 33; 16 8%), procedures (e.g., ports; *n* = 16; 4%), and rescue therapies (*n* = 2; 0%).

Six out of the eight MG treatment subcategories qualified for cross-platform analysis (Table 3). The remaining two subcategories (procedures: 11, 3% and Rescue therapies: 2, 1%) were not featured in posts from two or more distinct users in three or more of the participating groups and therefor did not qualify for cross-platform analysis. Topics of interest included: Side Effects (*n* = 129; 29%), Experience (*n* = 114; 26%), Efficacy (*n* = 76; 17%), Dosage (*n* = 52; 12%), Onset of effect (*n* = 31; 7%), Other (*n* = 30; 7%), and Duration of Action (*n* = 7; 2%). No individual side effects were validated via cross-platform analysis. Complete frequency tables for all question categories are included in the Supplementary material 3–9.

**Table 3:** Frequency of users posting MG treatment question subcategories validated using cross-platform analysis.

	*Group 1 users*	*Group 2 users*	*Group 3 users*	*Group 4 users*	*Unique users*	*Total users*	*Topic*
*MG Treatment*							
*Subcategory, n (%)*							
Symptomatic treatment	59 (20)	12 (24)	7 (19)	2 (25)	76 (95)	80 (100)	Side effects
Biologicals	59 (20)	13 (27)	9 (24)	2 (25)	76 (92)	83 (100)	Experience
IVIG (immunglobulins)	37 (13)	6 (12)	8 (22)	3 (38)	53 (98)	54 (100)	Side effects
General (non-specific)	34 (12)	6 (12)	6 (16)	1 (13)	45 (96)	47 (100)	Experience
Long-term oral-immunosuppressants	28 (10)	5 (10)	4 (11)	0 (0)	35 (96)	37 (100)	Side effects
Thymectomy	26 (9)	2 (4)	2 (5)	0 (0)	29 (97)	30 (100)	Experience
Total unique users	289 (100)	49 (100)	37 (100)	8 (100)	305 (80)	383 (100)	Side effects

% refers to the rate of users relative to the total number of users across each of the four groups. The unique user column refers to the total number of users minus repeat users who were observed posting the same question across multiple groups. MG-ADL, Myasthenia gravis activities of daily living (score).

Discussion of treatment across the participating groups was not limited to MG. One hundred and thirty three unique users asked 163 questions about non-MG treatment. The most common non-MG treatment questions concerned anaesthesia, which was asked about in 15 posts (9%), and antibiotics, which were discussed in nine posts (6%). No other non-MG medication, or group of non-MG medications, equalled ≥5% of the total questions in this category. Validation of these non-MG questions via cross-platform analysis was not possible as the majority of non-MG treatment discussion took place in Group 1 (80%).

**Figure 2:**
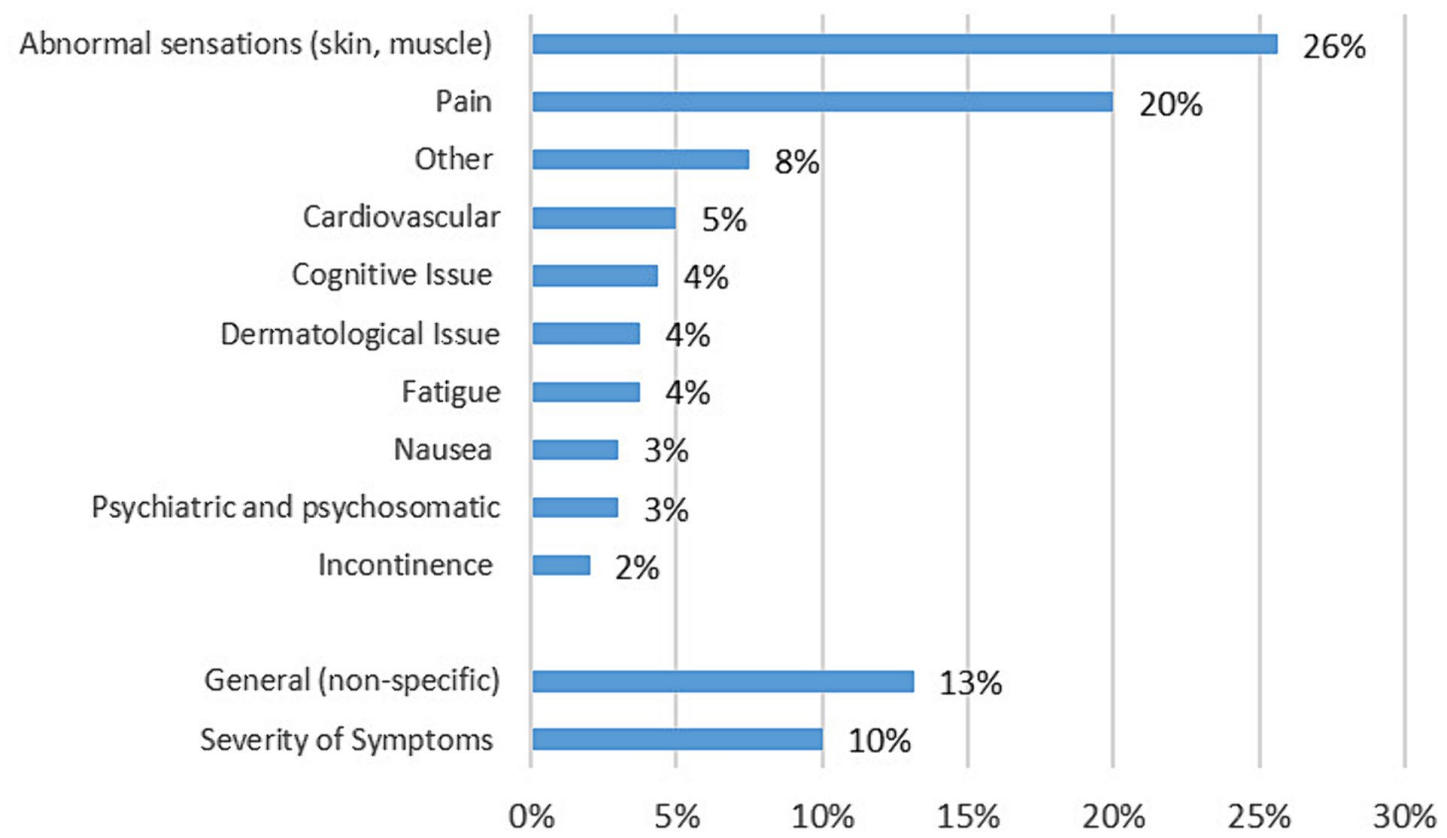
Frequency of Non-ADL symptom related questions observed across all groups.

Following MG treatment, the most popular category was symptom related queries. Two hundred and sixteen unique users asked 271 questions related to symptoms. Only 59% of symptom questions matched the items included in the standardised symptom assessment tool Myasthenia Gravis Activities of Daily living (MG-ADL). MG-ADL symptoms discussed included: breathing (*n* = 28; 25%), eyelid droop (*n* = 21; 19%) swallowing (*n* = 18; 16%), double vision (*n* = 16; 14%), impairment of ability to rise from a chair (*n* = 11; 10%), talking (*n* = 11; 10%), impairment of ability to brush or comb hair (*n* = 3; 3%), and chewing (*n* = 3; 3%). Non-MG-ADL symptom related questions included a number of specific symptoms and signs that could not be classified under the MG-ADL (see Figure 2). Full definitions of each Non-MG-ADL symptom is included in the accompanying material.

Only three MG-ADL symptom subcategories and two non-MG-ADL symptom subcategories qualified for cross-platform analysis (Table 4). Abnormal skin sensations were reported in 40 different posts and covered a wide variety of sensations including cramping, twitching, tingling, and spasms. The 32 posts featuring questions categorised as pain were less diverse. Although reports of pain were not limited to one region of the body, neck, back and shoulder pain accounted for 40% of the non-MG-ADL symptom questions and joint and muscle pain (13%).

**Table 4:** Frequency of users posting MG-ADL and Non-MG-ADL symptom subcategories that were validated using cross-platform analysis.

	*Group 1 users*	*Group 2 users*	*Group 3 users*	*Group 4 users*	*Unique users*	*Total users*
*ADL symptoms, n (%)*						
Breathing	20 (74)	5 (19)	2 (7)	0 (0)	25 (93)	27 (100)
Ptosis	14 (67)	2 (10)	5 (24)	0 (0)	21 (100)	21 (100)
Swallowing	11 (61)	2 (11)	3 (17)	2 (11)	17 (94)	18 (100)
*Non-ADL symptoms, n (%)*						
Abnormal sensations	28 (68)	6 (15)	3 (7)	4 (10)	39 (95)	41 (100)
Pain	23 (72)	6 (19)	3 (9)	0 (0)	32 (100)	32 (100)

% refers to the rate of users relative to the total number of users across each of the four groups. The unique user column refers to the total number of users minus repeat users who were observed posting the same question across multiple groups. MG-ADL, Myasthenia gravis activities of daily living (score).

Following the category “Symptoms,” “Living with MG” was the third most frequently category of question. One hundred and thirty eight unique users asked 174 questions categorised as such. This was the widest of all question categories due to the scope of issues, which could be classified as aspects of routine or daily life, namely: dietary (*n* = 53; 30%), Health Insurance (35; 20%), non-medical living assistance (e.g., mobility aids, prisms glasses) (*n* = 31; 18%), personal relationships (*n* = 12; 7%), mental health and stress management (*n* = 11; 6%), weather (*n* = 10; 6%), exercise (*n* = 9; 5%), employment (*n* = 6; 3%), travel and/or holiday making (*n* = 4; 2%), and driving (*n* = 3; 2%). Only three subcategories were validated using cross-platform analysis (Table 5).

**Table 5:** Frequency of users posting living with MG subcategories that were validated using cross-platform analysis.

	*Group 1 users*	*Group 2 users*	*Group 3 users*	*Group 4 users*	*Unique users*	*Total users*
*Living with MG*						
*Subcategory, n (%)*						
Dietary	36 (73)	4 (8)	7 (14)	2 (4)	48 (98)	49 (100)
Non-medical living assistance	23 (77)	4 (13)	2 (7)	1 (3)	29 (97)	30 (100)
Health insurance	20 (69)	5 (17)	2 (7)	2 (7)	28 (97)	29 (100)

% refers to the rate of users relative to the total number of users across each of the four groups. The unique user column is the total number of users minus repeat users who were observed posting the same question across multiple groups.

In total, 115 unique users asked 140 questions related to the diagnosis of MG (*n* = 76; 54%) or another disease (*n* = 64; 46%). Of the 57 unique users who asked about a non-MG diagnosis, the five most common non-MG diagnosis discussed were COVID-19 (*n* = 8; 14%), Thymoma (*n* = 5; 9%), Sleep Apnea (*n* = 3; 5%), Amyotrophic Lateral Sclerosis (ALS) (*n* = 3; 5%), Lambert-Eaton Syndrome (*n* = 3; 5%), and Amyotrophic Lateral Sclerosis (ALS) (*n* = 2; 4%). No non-MG diagnosis questions could be validated via cross-platform analysis.

Ninety unique users asked 110 questions related to tests. Test subcategories included; lab testing/ antibodies (44%); lung function (15%); nerve and muscle stimulation (15%); general unspecified (11%); imaging (6%); clinical examination (5%); and pathology (4%). With the exception of laboratory tests/antibodies, which asked about by 34 users in Group 1 (38%), six in Group 2 (7%), three in Group 3 (3%) and one in Group 4 (1%), no other question subcategory could be validated using cross-platform analysis.

Fifty seven unique users asked 62 questions concerning contacts in their local area. Of those users 73% (*n* = 42) sought information concerning professional contacts with MG experience in their local area, 18% sought other diagnosed patients and 9% enquired about lay and professional contacts. Only a need for professional contacts, which was asked about by 32 users in Group 1 (71%), eight in Group 2 (18%), and five in Group 3 (11%) was validated using cross-platform analysis. No requests for professional contacts were made in Group 4.

Fifty five questions asked by 44 unique users could not be categorised. As such, they could not be validated using cross-platform analysis and were excluded from further analysis. Due to data protection issues, it was not possible to include the exact wording of questions categorised as other.

## Discussion

The aim of this study was to better understand the needs of the MG population through the collation and categorisation of questions that users of MG social media asked fellow users on these platforms. The eight distinct question categories identified throughout the course of the observation period and later validated using cross-platform analysis convey a high need for information and willingness to share sensitive personal information including diagnosis, treatment and symptom related details order to gain insight.

This demand for information is not unique to the sample population. High need for information amongst those affected by MG was recently demonstrated in a study analysing emails written to a specialised treatment centre and patient organisation body (28). The results of the present study suggest that this need is not restricted to these official channels. Indeed, given that the disease effects approximately one in every 5,000 within the general population (2), the number of online resources identified in the sampling frame and unique users identified within the final sample population (*n* = 787) indicates that what is ostensibly a rare disease is not so rare online.

In drawing attention to this use, this study contributes to a growing body of literature investigating online health communities targeted at rare diseases (16, 17, 19, 29, 30): the results of which have shown that social media is an important information resource for many in the affected populations. MG appears to be no different in this regard. The four Facebook groups that made up the sample were utilised by a wide range of the affected population including the presumed diagnosed (83%), those seeking diagnosis (8%) and their support networks (9%). Regarding the latter, the 69 unique users identified as “Friends or Family” draws attention to the role of individual’s support networks—a topic which remains under-researched despite widespread acknowledgement of the debilitating nature of the disease and its impact upon employment (31) and caregivers (32, 33).

As for how different author categories in the sample population utilised these groups, the breadth and depth of the question categories and subcategories identified during the observation period further evidence the idea that the end of diagnostic “odyssey” (34) is the beginning of the therapeutic odyssey (35). In other words, the high need for information does not stop after diagnosis is received: it is a continual process. Within the sample population this was evident in the high frequency of presumed diagnosed users asking questions, and the nature of the questions discussed (see Table 2).

Regarding the nature of the questions asked, observed actively sharing what could be considered sensitive medical information with fellow group users in order users were to gain peer insight. This was most notable in questions related to MG treatment (31%) and Symptom information (19%). Whilst this openness and willingness to share sensitive information could be driven in part by limited access to expert diagnosis and treatment from experienced Neurologists (36) and/or a lack of trust in the medical profession stemming from interactions with healthcare providers who have little experience or knowledge of their condition (37, 38), both supply and demand factors must be taken into account when interpreting this behaviour. Previous studies have reported that those affected by MG are more likely to seek medical attention for concerning symptoms given their existent diseases status (39), and this is likely to extend to information sharing behaviours.

Reasoning aside, this study provides evidence that MG patients actively seek advice on range of topics, not all of which could be feasibly catered to by the medical profession. This was most notable in the “living with MG” category which covered issues related living assistance and insurance not within the neurologist’s remit. Practitioners should be able to point service users in the direction of reliable, professional, and objective information. Whilst the quality of information provided in response to the questions asked by the sample population is beyond the purview of this study, in lieu of transparent and accountable oversight mechanisms, it would not be appropriate to promote the use of these groups. Medical professionals should work instead with official patient organisations to tailor information to the needs of the diagnosed, undiagnosed and support networks of those effected by MG.

## Limitations

The primary limitation of this work is the representativeness of the sample. Although the cross-platform analysis allowed the researchers to attribute a higher level of credibility to the type of questions the sample population were interested in, the results cannot be generalised to the wider MG population. The results only reflect the concerns of the sample population during the observation period. Furthermore, the results do not provide insight into the reasons why some subjects are more or less common than others. With that said, the results provide an important insight into the practise of peer-to-peer support in a hard to reach population (40) using “naturalistic” user reported data (2, 19, 22). Moreover, by distinguishing between unique users, one-time users and repeat users, this study is able to differentiate between common interests held by multiple group users and individual user’s interests.

A secondary limitation of this study is the omission of engagement measures. Each post made on Facebook social media sites can be measured in terms of how other users interact it via “likes,” “shares,” and “comments.” Inclusion of this material was not possible due to the ephemeral nature of the content and resource limitations. As engagement with posts could be continual depending on the popularity of the post, such an approach would have required arbitrary cut offs for the measurement of engagement.

## Conclusion

Members of the MG population make active use of online health communities to seek and discuss practical information concerning various aspects of the disease, its diagnosis and care. The willingness of the sample population to share potentially sensitive personal information including diagnosis, treatment, and symptom related details conveys a high need for information not entirely catered to by the medical profession. Future studies should seek to further validate interest in the question categories identified herein by exploring the importance of these topics to the affected population in an offline setting.

## Data Availability

The raw data supporting the conclusions of this article will be made available by the authors, without undue reservation.
