# Supplementary material for: Myasthenia Gravis: utilising cross-platform quantitative content analysis to uncover and validate unmet needs
Source: Front Neurol. 2024 Sep 27;15:1474347. doi: 10.3389/fneur.2024.1474347 (PMC11483605; doi:10.3389/fneur.2024.1474347)
Supplement: Supplementary file 1 [file Table_1.docx]

## Supplement 1. Coding Scheme

| User Information | | | |
| --- | --- | --- | --- |
| Variable | Question | Coding Scheme | Description |
| v.1  Group Code | In which group was the post collected? | 1. (Group name Redacted) 2. (Group name Redacted) 3. (Group name Redacted) 4. (Group name Redacted) | Name of the group in which the post appears |
| v.2  Profile Code | Enter the users profile code | n/a | Use the Excel database, produce an encrypted profile code using the randomly generated 8-digit decryption key. |
| v.3  Date of Post | On What day of the observation process was the observation made? | Enter observation day (e.g. 1,2 etc.) | Day in which the observation was made. |
| V.4 Question | Does the post feature a question? | 1. Yes 2. No | Questions are defined as requests for information and were identified through the use of standard question words such as How, Who, What, When and Why and/or punctuation indicative of a question (?). |
| v.5  Gender | What is the poster’s sex/gender? | 1 – Male  2 – Female  3 – Unclear | Based on the user’s name and profile picture – approximate the users gender. |
| v.6  Author Information | What is the posters relation to MG? | 1. Presumed Diagnosed 2. Seeking Diagnosis 3. Friend/Family 4. Other | Based on the information contained within the text what is the diagnosis status of the author? |
| v.7  Author Info | If other please specify | Written answer – specify other. | Describe in short who the author is e.g. by profession. Do not quote phrases directly. |
| v.8  Route Post | How many questions are contain in the post. | Enter the number of questions featured in the post  e.g.  1-2-3-4-5 | If a post features multiple questions, enter the number the number of questions included. |
| v.9  Route post 2 | Which question is this | Enter which number of question this refers to e.g. question 1 (in the order they appear in the text) | Which question is this in relation to the route post? |

| Question Category | | | |
| --- | --- | --- | --- |
| Variable | Question | Coding Scheme | Explanation |
| v.10  Question Category | What type of question is being asked? | 1.Diagnosis  2. Living With MG  3. Location  4. Symptom  5. Test  6. MG Treatment  7. Non MG -treatment  8. Other | 1.Questions concerning Pathology/ Diagnosis  2. inquiries about aspects of living day to day life with mg  3. Request for local lay or professional contacts  4. Request concerning a specific symptom  5. Information requestion concerning a test/measure  6. Related to a specific MG treatment 7. Related to a non-MG treatment  8. everything else |

## Supplement 1. Coding Scheme (continued)

| Disease or Comorbidity | | | |
| --- | --- | --- | --- |
| **Variable** | Question | Coding Scheme | Explanation |
| v.11 Diagnosis  Question Category | What type of question is being asked? | 1. Diagnosis of MG 2. Diagnosis of a comorbidity | 1. MG Pathology/ diagnosis question 2. Non-MG Pathology/ diagnosis question |
| v.12 Diagnosis 2 | What specific diagnosis is being discussed? | Written answer – if not MG what condition is being discussed? | What is being discussed, e.g covid. Do not quote phrases directly. |

| Living with MG | | | |
| --- | --- | --- | --- |
| Variable | Question | Coding Scheme | Explanation |
| v.13 Living with MG  Question Category | What type of question is being asked? | 1. Dietary 2. Exercise 3. Living Assistance 4. Employment 5. Relationships/ Social activity 6. Insurance 7. Weather 8. Mental Health and Stress Management 9. Driving 10. Travel/ Holiday Making | 1. Questions concerning diet 2. Questions concerning physical activity/ exercise. 3. Questions concerning support or care. 4. Questions related to formal paid work 5. Questions related to social relationships or activities 6. Insurance based questions 7. Weather/ Temperature related questions 8. Questions related to mental health and stress management. 9. Questions concerning the driving of vehicles. 10. Questions concerning holidays and/or travel. |
| v.14 LWMG Question 2 | What specific aspect of living with MG is being discussed? | Written answer – what aspect of living of MG is being discussed? | Describe in short what is being discussed. Do not quote phrases directly. |

| Location | | | |
| --- | --- | --- | --- |
| **Variable** | Question | Coding Scheme | Explanation |
| v.15 Location  Question Category | What type of location question is being asked? | 1.Lay contacts  2.Professional Contacts  3.Lay and Professional Contacts | 1. Requests for non-health professional contacts  2. Requests for professional contacts including, doctors, medical centres, and hospitals  3. Requests featuring both of the above. |

## Supplement 1. Coding Scheme (continued)

| **Test** | | | |
| --- | --- | --- | --- |
| **Variable** | Question | Coding Scheme | Explanation (examples) |
| v.19 Test  Question Category | What Test is being asked about? | 1. Laboratory findings 2. electrophysiological testing 3. Pathology 4. Imaging 5. Lung Function 6. Clinical Examination 7. General/ Unspecified | 1.Antibodies test, bloodwork, acetylcholine receptor (AChR), non-specified lab results.  2. Electromyograph (EMG), Repetitive Nerve Stimulation  3. Biopsy  4. Magnetic Resonance Imaging (MRI)  5. Breath test, Pulmonary Function test  6. Ice pack test, clinical exam  7. General or unspecified questions about tests |
| v.20 Test Question 2 | What specific test is being discussed? | Written answer – what test is being discussed? | Describe in short what is being discussed. Do not quote phrases directly. |

| Treatment | | | |
| --- | --- | --- | --- |
| Variable | Question | Coding Scheme | Explanation (examples) |
| v.22 Treatment  Question Category | What Treatment Category is being asked about? | 1. Symptomatic treatment 2. Corticosteroids. 3. Long term immunosuppressants 4. IVIG (Immunoglobulins) 5. Rescue Therapy 6. Biologicals 7. Thymectomy 8. Process, procedure (e.g. port) 9. General. | 1.Pyridostigmin, Kalymin, Mestinon  2. Prednisone, Prednisolone, steroids  3. (Azathioprin (bn: Imurek,...), Mycophenolatmofetil (bn: Cellcept, Mowel,...)  4. IVIG (immunglobulins)  5. PLEX, plasmapherisus, plasmaexchange immunoabsortoion  6. eculizumab (Soliris), Ravulizumab (Ultomiris), efgartigimod (vyvgart)  7. Thymectomy  8. Ports, Trachs  9. Non-specific treatment question that do no fit into other categories. |
| v.23 Treatment  Question Category | What specific treatment is being discussed? | Written answer – what symptom is being discussed? | Describe in short what is being discussed. Do not quote phrases directly. |
| v.25 Treatment Question 2 | Why is it being discussed? Select only one. | 1. Side Effects 2. Efficacy 3. dosage 4. Duration of Action 5. Onset of Effect 6. Experience 7. Other | 1. adverse reactions  2. ability to produce intended result.  3. the size or frequency of dose  4. length of effect  5. time to work  6. non-specific calls for personal experience.  7. Anything else |
| V. 26 Side Effect | If a side effect is mentioned what side effect. | Written answer – what test is being discussed? | Describe in short what is being discussed. No direct quotation. |

| Other | | |
| --- | --- | --- |
| Variable | Question | Coding Scheme |
| v.27  Other  Question Category | What type of question is being asked? | Written response - Provide a specific account of the question being asked. No direct quotation.  FLAG with co-researcher. |

## Supplement 2. Sampling frame search strategy

The search strategy utilised a two-stage approach in order to identify relevant forums. First a Google and Social Media Platform Search was conducted. The Social Media Platforms searched included Facebook and Reddit. These platforms were selected due to their format and prominence (number of monthly active users and type of content hosted: primarily text).

The second search strategy used focused on Official Myasthenia Gravis Organisations/ Charities Websites. This was carried out in order to identify any forums/ groups which had an official backing. Relevant Organisations and Charities were identified through membership of European Association of Myasthenia Gravis Patients Associations (EUMGA). The list of members can be found on the respective organisations’ websites. The United Kingdom and United States of America were not included in the listed members of the EUMGA and thus relevant bodies were searched for using a google search.

The results of the preliminary research into forums found 80 Facebook Groups concerning Myasthenia Gravis in Humans. Two Facebook groups were identified as concerning Myasthenia Gravis in Dogs. A full list of groups included in the sampling frame is available upon request. Four other platforms were identified as hosts of myasthenia gravis forums including Myasthenia-Gravis.com; Neurotalk; DailyStrength and Reddit. A full list of groups included in the sampling frame is available upon request.

## **Supplement 3.** MG treatment question frequencies

**Table 6.** Frequency of users posting Myasthenia gravis treatment questions.

**Table 7.** Myasthenia gravis treatment question multiple group users

|  | *Group 1  Users* | *Group 2  Users* | *Group 3 Users* | *Group 4 Users* | *Unique Users* | *Total  Users* |
| --- | --- | --- | --- | --- | --- | --- |
| ***MG Treatment***  ***Subcategory, n (%)*** | | | | | | |
| 1. Symptomatic treament | 59 (73.75) | 12 (15.00) | 7 (8.75) | 2 (2.50) | 76 (95.00) | 80 (100) |
| 2. Corticosteroids | 33 (84.62) | 5 (12.82) | 1 (2.56) | 0 (0.00) | 38 (97.44) | 34 (100) |
| 3. Long-term oral immunosuppressants | 28 (75.68) | 5 (13.51) | 4 (10.81) | 0 (0.00) | 35 (94.59) | 37 (100) |
| 4. IVIG (immunglobulins) | 37 (68.52) | 6 (11.11) | 8 (14.81 | 3 (5.56) | 53 (98.15) | 54 (100) |
| 5. Rescue-Therapy | 2 (100.00) | 0 (0.00) | 0 (0.00) | 0 (0.00) | 2 (100) | 2 (100) |
| 6. Biologicals | 59 (71.08) | 13 (10.84) | 9 (10.84) | 2 (2.41) | 76 (91.57) | 83 (100) |
| 7. Thymectomy | 26 (86.67) | 2 (6.67) | 2 (6.67) | 0 (0.00) | 29 (96.67) | 30 (100) |
| 8. Procedures (e.g port) | 11 (100) | 0 (0.00) | 0 (0.00) | 0 (0.00) | 11 (100) | 11 (100) |
| 9. General (or multiple) | 34 (72.34) | 6 (12.77) | 6 (12.7) | 1 (2.13) | 45 (95.74) | 46 (100) |
| Total Users | 289 (75.45) | 44 (11.48) | 37 (9.66) | 7 (1.82) | 305 (79.63) | 383 (100) |

| *Category* | *Multiple Groups Users* | *Groups Used* |
| --- | --- | --- |
| 1. | 3 Users | User 1 – 1,3  User 2 – 1,3,4 User 3 – Groups 1,4 |
| 2. | 1 User | User 1 – Groups 1,2 |
| 3. | 2 Users | User 1 – Groups 1,3  User 2 – Groups 1,2 |
| 4. | 1 User | User 1 – Groups 1,3 |
| 6. | 5 Users | User 1 - Groups - 1,2,3  User 2 – Groups 2,3  User 3 – Groups 1,3,4 User 4 – Groups 1,3  User 5 – Groups 1,2 |
| 7. | 1 User | User 1 – Groups 1,3 |
| 9. | 1 User | User 1 - Groups - 1,2,3 |

Note : % refers to the rate of users relative to the total number of users across each of the four groups. Total users is the total number of users observed across all four groups. The total number of unique users does not equal the users displayed due to the presence of unique users who participated across multiple groups. Long-term oral immunosuppressants are non-cortocosteroid.

Note: this table depicts users of multiple groups who asked the same type of question across multiple groups. Abbreviation: MG-ADL Myasthenia gravis activities of daily living (score)

## **Supplement 4.** MG-ADL symptom question frequencies

**Table 8.** Frequency of users posting MG-ADL symptom questions.

**Table 9.** MG-ADL Symptom question multiple group users

.

|  | *Group 1  Users* | *Group 2  Users* | *Group 3 Users* | *Group 4 Users* | *Unique Users* | *Total  Users* |
| --- | --- | --- | --- | --- | --- | --- |
| ***ADL Symptoms, n (%)*** | | | | | | |
| 1. Talking | 7 (63.64) | 3 (27.27) | 0 (0.00) | 1 (9.09) | 11 (100) | 11 (100) |
| 2. Chewing | 2 (66.67) | 0 (0.00) | 0 (0.00) | 1 (33.33) | 3 (100) | 3 (100) |
| 3. Swallowing | 11 (61.11) | 2 (11.11) | 3 (16.67) | 2 (11.11) | 17 (94.44) | 18 (100) |
| 4. Breathing | 20 (74.07) | 5 (18.52) | 2 (7.41) | 0 (0.00) | 25 (92.59 | 27 (100) |
| 5. Impairment of ability to brush or comb hair | 0 (0.00) | 3 (100) | 0 (0.00) | 0 (0.00) | 3 (100) | 3 (100) |
| 6. Impairment of ability to rise from a chair | 7 (70.00) | 2 (20.00) | 1 (10.00) | 0 (0.00) | 10 (100) | 10 (100) |
| 7. Double vision | 1 (25.00) | 1 (25.00) | 2 (50.00) | 0 (0.00) | 4 (100) | 4 (100) |
| 8. Eyelid droop | 14 (66.67) | 2 (9.52) | 5 (23.81) | 0 (0.00) | 21 (100) | 21 (100) |
| Total Users | 62 (63.92) | 18 (18.56) | 13 (13.40) | 4 (4.12) | 97 (100) | 97 (100) |

| *Category* | *Multiple Groups Users* | *Groups Used* |
| --- | --- | --- |
| 3. | 1 User | User 1 – Groups 3,4 |
| 4. | 2 Users | User 1 – Groups 1,3  User 2 – Groups 1,3 |

Note: this table depicts users of multiple groups who asked the same type of question across multiple groups. Abbreviation: MG-ADL Myasthenia gravis activities of daily living (score)

Note : % refers to the rate of users relative to the total number of users across each of the four groups. Total users is the total number of users observed across all four groups. The total number of unique users does not equal the users displayed due to the presence of unique users who participated across multiple groups. Abbreviation: MG-ADL Myasthenia gravis activities of daily living (score)

## **Supplement 5.** Non-MG-ADL symptom question frequencies

**Table 10.** Frequency of Users posting non-MG-ADL symptom questions

|  | *Group 1  Users* | *Group 2  Users* | *Group 3 Users* | *Group 4 Users* | *Unique Users* | *Total  Users* |
| --- | --- | --- | --- | --- | --- | --- |
| ***Non-MG- ADL  Symptoms, n (%)*** | | | | | | |
| 1. Abnormal sensations | 28 (68.29) | 6 (14.63) | 3 (7.32) | 4 (9.76) | 39 (95.12) | 41 (100) |
| 2. Pain | 23 (71.88) | 6 (18.75) | 3 (9.38) | 0 (0.00) | 32 (100) | 32 (100) |
| 3. General (non-specific) | 13 (65.00) | 5 (25.00) | 2 (10.00) | 0 (0.00) | 19 (95.00) | 20 (100) |
| 4. Severity of Symptoms | 15 (93.75) | 1 (6.25) | 0 (0.00) | 0 (0.00) | 16 (100) | 16 (100) |
| 5. Cognitive Issue | 6 (85.71) | 0 (0.00) | 1 (14.29) | 0 (0.00) | 7 (100) | 7 (100) |
| 6. Fatigue | 1 (16.67) | 5 (83.33) | 0 (0.00) | 0 (0.00) | 6 (100) | 6 (100) |
| 7. Dermatalogical Issue | 3 (50.00) | 1 (16.67) | 2(33.33) | 0 (0.00) | 6 (100) | 6 (100) |
| 8. Cardiovascular | 5 (71.43) | 2 (28.57) | 0 (0.00) | 0 (0.00) | 7 (100) | 7 (100) |
| 9. Psychiatric and psychosomatic | 2 (50.00) | 2 (50.00) | 0 (0.00) | 0 (0.00) | 4 (100) | 4 (100) |
| 10. Incontinence | 0 (0.00) | 2 (66.67) | 1 (33.33) | 0 (0.00) | 2 (66.37) | 3 (100) |
| 11. Nausea | 2 (50.00) | 2 (50.00) | 0 (0.00) | 0 (0.00) | 4 (100) | 4 (100) |
| 12. Other | 10 (83.33) | 2 (16.77) | 0 (0.00) | 0 (0.00) | 12 (100) | 12 (100) |
| Total Users | 108(68.35) | 34 (21.52) | 12 (7.59) | 4 (2.53) | 138 (87.34) | 158 (100) |

**Table 11.** Non-MG-ADL Symptom question multiple group users

.

Note: this table depicts users of multiple groups who asked the same type of question across multiple groups. Abbreviation: MG-ADL Myasthenia gravis activities of daily living (score)

| *Category* | *Multiple Groups Users* | *Groups Used* |
| --- | --- | --- |
| 1. | 1 User | 1 User – Group 1,3,4 |
| 3. | 1 User | 1 User – Group 1,2 |
| 10. | 1 User | 1 User – Group 2,3 |

Note : % refers to the rate of users relative to the total number of users across each of the four groups. Total users is the total number of users observed across all four groups. The total number of unique users does not equal the users displayed due to the presence of unique users who participated across multiple groups. Abbreviation: MG-ADL Myasthenia gravis activities of daily living (score)

## **Supplement 6.** Test question frequencies

**Table 13.** - Test question multiple group users

**Table 12.** - Frequency of Users posting test questions

|  | *Group 1  Users* | *Group 2  Users* | *Group 3 Users* | *Group 4 Users* | *Unique Users* | *Total  Users* |
| --- | --- | --- | --- | --- | --- | --- |
| ***Tests, n (%)*** | | | | | | |
| 1. Lab testing/ Antibodies | 34 (77.27) | 6 (13.64) | 3 (6.82) | 1 (2.27) | 41 (93.18) | 44 (100) |
| 2. Nerve and Muscle Stimulation | 14 (87.50) | 1 (6.25) | 1 (6.25) | 0 (0.00) | 15 (93.75) | 16 (100) |
| 3. Pathology | 4 (100.00) | 0 (0.00) | 0 (0.00) | 0 (0.00) | 4 (100) | 4 (100) |
| 4. Imaging | 6 (85.71) | 0 (0.00) | 1 (14.29) | 0 (0.00) | 7 (100) | 7 (100) |
| 5. Lung Function | 14 (93.33) | 1 (6.67) | 0 (0.00) | 0(0.00) | 15 (100) | 15 (100) |
| 6. Clinical Examination | 5 (100.00) | 0 (0.00) | 0 (0.00) | 0 (0.00) | 5 (100) | 5 (100) |
| 7. General Unspecified | 9 (75.00) | 3 (25.00) | 0 (0.00) | 0 (0.00) | 11 (91.67) | 12 (100) |
| Total Users | 86 (83.50) | 11 (10.68) | 5 (4.85) | 1 (0.97) | 90 (87.37) | 103 (100) |

| *Category* | *Multiple Groups Users* | *Groups Used* |
| --- | --- | --- |
| 1. | 2 User | User 1 – Groups 1,3  User 2 – Groups 1, 2, 4 |
| 3. | 1 Users | User 1 – Groups 1,3 |
| 10. | 1 User | User 1 – Group 1,2 |

Note: this table depicts users of multiple groups who asked the same type of question across multiple groups.

Note : % refers to the rate of users relative to the total number of users across each of the four groups. Total users is the total number of users observed across all four groups. The total number of unique users does not equal the users displayed due to the presence of unique users who participated across multiple groups.

## **Supplement 7.** Location question frequencies

**Table 15.** Location question multiple group users

**Table 14.** - Frequency of Users posting location questions

Note: this table depicts users of multiple groups who asked the same type of question across multiple groups.

|  | *Group 1  Users* | *Group 2  Users* | *Group 3 Users* | *Group 4 Users* | *Unique Users* | *Total  Users* |
| --- | --- | --- | --- | --- | --- | --- |
| ***Location  questions, n (%)*** | | | | | | |
| Lay Contacts | 5 (45.45) | 1 (9.09) | 4 (36.36) | 1 (9.09) | 11 (100) | 11 (100) |
| Professional Contacts | 32 (72.73) | 7 (15.91) | 5 (11.36) | 0 (0.00) | 42 (95.45) | 44 (100) |
| Lay and  Professional Contacts | 5 (100) | 0 (0.00) | 0 (0.00) | 0 (0.00) | 5 (100) | 5 (100) |
| Total users | 42 (70.00) | 8 (13.33) | 9 (15.00) | 1 (1.67) | 57 (95.00) | 60 (100) |

| *Category* | *Multiple Groups Users* | *Groups Used* |
| --- | --- | --- |
| Lay Contacts | 1 User | User 1 – Groups 1,4 |
| Professional Contacts | 2 Users | User 1 – Groups 1,3 User 2 – Groups 2,3 |

Note: % refers to the rate of users relative to the total number of users across each of the four groups. Total users is the total number of users observed across all four groups. The total number of unique users does not equal the users displayed due to the presence of unique users who participated across multiple groups.

## **Supplement 8.** Non-Myasthenia Gravis treatment question frequencies

**Table 16.** - Frequency of Users posting Non-MG-Treatment questions

|  | Group 1 Users | Group 2 Users | Group 3 Users | Group4 Users | Unique Users | Total  Users |
| --- | --- | --- | --- | --- | --- | --- |
| *Non-MG-Treatment, n (%)* | | | | | | |
| Anasthesia | 15 (100) | 0 (0.00) | 0 (0.00) | 0 (0.00) | 15 (100) | 15 (100) |
| Antibiotics | 6 (75.00) | 2 (25.00) | 0 (0.00) | 0 (0.00) | 8 (100) | 8 (100) |
| List of medications not to take | 2 (50.00) | 1 (25.00) | 1 (25.00) | 0 (0.00) | 4 (100) | 4 (100) |
| Antihistamines | 3 (100) | 0 (0.00) | 0 (0.00) | 0 (0.00) | 3 (100) | 3 (100) |
| Naltrexone | 3 (100) | 0 (0.00) | 0 (0.00) | 0 (0.00) | 3 (100) | 3 (100) |
| Ozempic | 1 (75.00) | 1 (12.25) | 1 (12.25) | 0 (0.00) | 3 (100) | 3 (100) |
| Alburetol | 2 (100) | 0 (0.00) | 0 (0.00) | 0 (0.00) | 2 (100) | 2 (100) |
| Anti-depressents | 0 (0.00) | 2 (100) | 0 (0.00) | 0 (0.00) | 2 (100) | 2 (100) |
| Botox Injections | 2 (100) | 0 (0.00) | 0 (0.00) | 0 (0.00) | 2 (100) | 2 (100) |
| Cold medication (unspecified) | 2 (100) | 0 (0.00) | 0 (0.00) | 0 (0.00) | 2 (100) | 2 (100) |
| Continuous positive airway pressure (CPAP) | 2 (100) | 0 (0.00) | 0 (0.00) | 0 (0.00) | 2 (100) | 2 (100) |
| Doxycycline | 2 (100) | 0 (0.00) | 0 (0.00) | 0 (0.00) | 2 (100) | 2 (100) |
| Gabapanetin | 2 (100) | 0 (0.00) | 0 (0.00) | 0 (0.00) | 2 (100) | 2 (100) |
| Homeopathic remedies (unspecified) | 2 (100) | 0 (0.00) | 0 (0.00) | 0 (0.00) | 2 (100) | 2 (100) |
| Multiple | 2 (100) | 0 (0.00) | 0 (0.00) | 0 (0.00) | 2 (100) | 2 (100) |
| Period | 2 (100) | 0 (0.00) | 0 (0.00) | 0 (0.00) | 2 (100) | 2 (100) |
| Propantheline | 0 (0.00) | 2 (100) | 0 (0.00) | 0 (0.00) | 2 (100) | 2 (100) |
| Rheumatologist | 2 (100) | 0 (0.00) | 0 (0.00) | 0 (0.00) | 2 (100) | 2 (100) |
| Sleep medication (unspecified) | 1 (50.00) | 1 (50.00) | 0 (0.00) | 0 (0.00) | 2 (100) | 2 (100) |
| Statin | 2 (100) | 0 (0.00) | 0 (0.00) | 0 (0.00) | 2 (100) | 2 (100) |
| Stella Ganglion Block | 2 (100) | 0 (0.00) | 0 (0.00) | 0 (0.00) | 2 (100) | 2 (100) |
| Surgery (unspecified) | 1 (50.00) | 1 (50.00) | 0 (0.00) | 0 (0.00) | 2 (100) | 2 (100) |
| Ventilator | 2 (100) | 0 (0.00) | 0 (0.00) | 0 (0.00) | 2 (100) | 2 (100) |
| Yellow fever vaccine | 1 (50.00) | 1 (50.00) | 0 (0.00) | 0 (0.00) | 2 (100) | 2 (100) |

## **Supplement 8.** Non-Myasthenia Gravis treatment question frequencies (continued)

**Table 16.** - Frequency of Users posting Non-MG-Treatment questions

|  | Group 1 Users | Group 2 Users | Group 3 Users | Group4 Users | Unique Users | | Total  Users |
| --- | --- | --- | --- | --- | --- | --- | --- |
| *Non-MG-Treatment, n (%)* | | | | | | | |
| Acupuncture | 1 (100) | 0 (0.00) | 0 (0.00) | 0 (0.00) | 1 (100) | 1 (100) | |
| Advair | 1 (100) | 0 (0.00) | 0 (0.00) | 0 (0.00) | 1 (100) | 1 (100) | |
| Ahernia Surgery | 1 (100) | 0 (0.00) | 0 (0.00) | 0 (0.00) | 1 (100) | 1 (100) | |
| Albuterol | 0 (0.00) | 0 (0.00) | 1 (100) | 0 (0.00) | 1 (100) | 1 (100) | |
| Antipsychotics (unspecified) | 1 (100) | 0 (0.00) | 0 (0.00) | 0 (0.00) | 1 (100) | 1 (100) | |
| Antiacid | 1 (100) | 0 (0.00) | 0 (0.00) | 0 (0.00) | 1 (100) | 1 (100) | |
| Anxiety Medication (unspecified) | 0 (0.00) | 0 (0.00) | 1 (100) | 0 (0.00) | 1 (100) | 1 (100) | |
| Apeitite suppressents (unspecified) | 1 (100) | 0 (0.00) | 0 (0.00) | 0 (0.00) | 1 (100) | 1 (100) | |
| Atorvastin | 1 (100) | 0 (0.00) | 0 (0.00) | 0 (0.00) | 1 (100) | 1 (100) | |
| Bactrim | 0 (0.00) | 0 (0.00) | 0 (0.00) | 1 (0.00) | 1 (100) | 1 (100) | |
| Bariatric Sleeve | 0 (0.00) | 0 (0.00) | 0 (0.00) | 0 (0.00) | 1 (100) | 1 (100) | |
| Benfotiamine | 0 (0.00) | 1 (100) | 0 (0.00) | 0 (0.00) | 1 (100) | 1 (100) | |
| Bilevel positive airway pressure (Bpap) | 1 (100) | 0 (0.00) | 0 (0.00) | 0 (0.00) | 1 (100) | 1 (100) | |
| Buscopan | 1 (100) | 0 (0.00) | 0 (0.00) | 0 (0.00) | 1 (100) | 1 (100) | |
| Buspirone | 1 (100) | 0 (0.00) | 0 (0.00) | 0 (0.00) | 1 (100) | 1 (100) | |
| Cervical spine surgery | 1 (100) | 0 (0.00) | 0 (0.00) | 0 (0.00) | 1 (100) | 1 (100) | |
| Chemo Therapy | 1 (100) | 0 (0.00) | 0 (0.00) | 0 (0.00) | 1 (100) | 1 (100) | |
| Clonezepan | 1 (100) | 0 (0.00) | 0 (0.00) | 0 (0.00) | 1 (100) | 1 (100) | |
| Colchicine | 1 (100) | 0 (0.00) | 0 (0.00) | 0 (0.00) | 1 (100) | 1 (100) | |
| Cold medication | 1 (100) | 0 (0.00) | 0 (0.00) | 0 (0.00) | 1 (100) | 1 (100) | |
| Contrast therapy | 0 (0.00) | 0 (0.00) | 1 (100) | 0 (0.00) | 1 (100) | 1 (100) | |
| Cortisone | 1 (100) | 0 (0.00) | 0 (0.00) | 0 (0.00) | 1 (100) | 1 (100) | |
| Cough medicine | 1 (100) | 0 (0.00) | 0 (0.00) | 0 (0.00) | 1 (100) | 1 (100) | |
| Covid Booster | 0 (0.00) | 0 (0.00) | 1 (100) | 0 (0.00) | 1 (100) | 1 (100) | |
| Covid Medication (unspecified) | 1 (100) | 0 (0.00) | 0 (0.00) | 0 (0.00) | 1 (100) | 1 (100) | |
| Covixyl | 1 (100) | 0 (0.00) | 0 (0.00) | 0 (0.00) | 1 (100) | 1 (100) | |
| Cramp Relief Medication (unspecified) | 1 (100) | 0 (0.00) | 0 (0.00) | 0 (0.00) | 1 (100) | 1 (100) | |
| Cyclosporine | 0 (0.00) | 0 (0.00) | 0 (0.00) | 1 (0.00) | 1 (100) | 1 (100) | |

|  | Group 1 Users | Group 2 Users | Group 3 Users | Group4 Users | Unique Users | Total  Users |
| --- | --- | --- | --- | --- | --- | --- |
| *Non-MG-Treatment, n (%)* | | | | | | |
| Eyelid surgery | 1 (100) | 0 (0.00) | 0 (0.00) | 0 (0.00) | 1 (100) | 1 (100) |
| Firdapse | 1 (100) | 0 (0.00) | 0 (0.00) | 0 (0.00) | 1 (100) | 1 (100) |
| Gabapentin | 0 (0.00) | 0 (0.00) | 1 (100) | 0 (0.00) | 1 (100) | 1 (100) |
| Glycopyrrolate | 1 (100) | 0 (0.00) | 0 (0.00) | 0 (0.00) | 1 (100) | 1 (100) |
| homeopathic remedies | 1 (100) | 0 (0.00) | 0 (0.00) | 0 (0.00) | 1 (100) | 1 (100) |
| Hydrocodone | 1 (100) | 0 (0.00) | 0 (0.00) | 0 (0.00) | 1 (100) | 1 (100) |
| Hydroxychloroquine | 1 (100) | 0 (0.00) | 0 (0.00) | 0 (0.00) | 1 (100) | 1 (100) |
| Imiquimod | 0 (0.00) | 1 (100) | 0 (0.00) | 0 (0.00) | 1 (100) | 1 (100) |
| Inhaler | 0 (0.00) | 0 (0.00) | 0 (0.00) | 0 (0.00) | 1 (100) | 1 (100) |
| Knee replacement surgery | 1 (100) | 0 (0.00) | 0 (0.00) | 0 (0.00) | 1 (100) | 1 (100) |
| Lip Fillers | 1 (100) | 0 (0.00) | 0 (0.00) | 0 (0.00) | 1 (100) | 1 (100) |
| Lyrica | 0 (0.00) | 1 (100) | 0 (0.00) | 0 (0.00) | 1 (100) | 1 (100) |
| Mental health evaluation | 1 (100) | 0 (0.00) | 0 (0.00) | 0 (0.00) | 1 (100) | 1 (100) |
| Migraine Relief General | 0 (0.00) | 1 (100) | 0 (0.00) | 0 (0.00) | 1 (100) | 1 (100) |
| Mobic | 1 (100) | 0 (0.00) | 0 (0.00) | 0 (0.00) | 1 (100) | 1 (100) |
| Morphine patch | 0 (0.00) | 1 (100) | 0 (0.00) | 0 (0.00) | 1 (100) | 1 (100) |
| Mucinex | 1 (100) | 0 (0.00) | 0 (0.00) | 0 (0.00) | 1 (100) | 1 (100) |
| Muscle relaxers | 0 (0.00) | 0 (0.00) | 1 (100) | 0 (0.00) | 1 (100) | 1 (100) |
| Mycophenolate | 1 (100) | 0 (0.00) | 0 (0.00) | 0 (0.00) | 1 (100) | 1 (100) |
| Myrbetriq | 1 (100) | 0 (0.00) | 0 (0.00) | 0 (0.00) | 1 (100) | 1 (100) |
| Negative Inspirartory Force Meter | 0 (0.00) | 0 (0.00) | 1 (100) | 0 (0.00) | 1 (100) | 1 (100) |
| Nerve block procedure | 1 (100) | 0 (0.00) | 0 (0.00) | 0 (0.00) | 1 (100) | 1 (100) |
| Neuro Muscular Blocking Agents (unspecified) | 1 (100) | 0 (0.00) | 0 (0.00) | 0 (0.00) | 1 (100) | 1 (100) |
| Nicotinamide adenine dinucleotide | 1 (100) | 0 (0.00) | 0 (0.00) | 0 (0.00) | 1 (100) | 1 (100) |
| Ocular surgery | 1 (100) | 0 (0.00) | 0 (0.00) | 0 (0.00) | 1 (100) | 1 (100) |
| Otelza | 1 (100) | 0 (0.00) | 0 (0.00) | 0 (0.00) | 1 (100) | 1 (100) |
| Oxygen concentrator | 1 (100) | 0 (0.00) | 0 (0.00) | 0 (0.00) | 1 (100) | 1 (100) |
|  |  |  |  |  |  |  |

## **Supplement 8.** Non-Myasthenia Gravis treatment question frequencies (continued)

**Table 16.** - Frequency of Users posting Non-MG-Treatment questions

## **Supplement 8.** Non-Myasthenia Gravis treatment question frequencies (continued)

**Table 16** - Frequency of Users posting Non-MG-Treatment questions

|  | Group 1 Users | Group 2 Users | Group 3 Users | Group4 Users | Unique Users | Total  Users |
| --- | --- | --- | --- | --- | --- | --- |
| *Non-MG-Treatment, n (%)* | | | | | | |
| Palliative care | 0 (0.00) | 1 (100) | 0 (0.00) | 0 (0.00) | 1 (100) | 1 (100) |
| Paxlovid | 1 (100) | 0 (0.00) | 0 (0.00) | 0 (0.00) | 1 (100) | 1 (100) |
| Physical Therapy | 1 (100) | 0 (0.00) | 0 (0.00) | 0 (0.00) | 1 (100) | 1 (100) |
| Pinex or reeses | 1 (100) | 0 (0.00) | 0 (0.00) | 0 (0.00) | 1 (100) | 1 (100) |
| Pregnancy Supplements | 1 (100) | 0 (0.00) | 0 (0.00) | 0 (0.00) | 1 (100) | 1 (100) |
| Prevagen | 1 (100) | 0 (0.00) | 0 (0.00) | 0 (0.00) | 1 (100) | 1 (100) |
| Propanthaline | 0 (0.00) | 1 (100) | 0 (0.00) | 0 (0.00) | 1 (100) | 1 (100) |
| Provigil | 1 (100) | 0 (0.00) | 0 (0.00) | 0 (0.00) | 1 (100) | 1 (100) |
| Pyramine | 1 (100) | 0 (0.00) | 0 (0.00) | 0 (0.00) | 1 (100) | 1 (100) |
| Radium | 1 (100) | 0 (0.00) | 0 (0.00) | 0 (0.00) | 1 (100) | 1 (100) |
| ResMed Astral 150 | 1 (100) | 0 (0.00) | 0 (0.00) | 0 (0.00) | 1 (100) | 1 (100) |
| Ritalin | 1 (100) | 0 (0.00) | 0 (0.00) | 0 (0.00) | 1 (100) | 1 (100) |
| Robinul (glycopyrrolate) | 1 (100) | 0 (0.00) | 0 (0.00) | 0 (0.00) | 1 (100) | 1 (100) |
| Rocethin | 1 (100) | 0 (0.00) | 0 (0.00) | 0 (0.00) | 1 (100) | 1 (100) |
| Sea sickness Medication (unspecified) | 1 (100) | 0 (0.00) | 0 (0.00) | 0 (0.00) | 1 (100) | 1 (100) |
| Shingles vaccine | 1 (100) | 0 (0.00) | 0 (0.00) | 0 (0.00) | 1 (100) | 1 (100) |
| Osteoporosis medication (unspecified) | 0 (0.00) | 0 (0.00) | 1 (100) | 0 (0.00) | 1 (100) | 1 (100) |
| Singulair | 1 (100) | 0 (0.00) | 0 (0.00) | 0 (0.00) | 1 (100) | 1 (100) |
| Therapy/ Mental Health | 0 (0.00) | 1 (100) | 0 (0.00) | 0 (0.00) | 1 (100) | 1 (100) |
| Topical Analgesics | 1 (100) | 0 (0.00) | 0 (0.00) | 0 (0.00) | 1 (100) | 1 (100) |
| Tramadol | 1 (100) | 0 (0.00) | 0 (0.00) | 0 (0.00) | 1 (100) | 1 (100) |
| Tylenol | 1 (100) | 0 (0.00) | 0 (0.00) | 0 (0.00) | 1 (100) | 1 (100) |
| Vancomycin | 1 (100) | 0 (0.00) | 0 (0.00) | 0 (0.00) | 1 (100) | 1 (100) |
| Vertebreoplasty | 1 (100) | 0 (0.00) | 0 (0.00) | 0 (0.00) | 1 (100) | 1 (100) |
| Vicodin | 1 (100) | 0 (0.00) | 0 (0.00) | 0 (0.00) | 1 (100) | 1 (100) |
| Zetia | 1 (100) | 0 (0.00) | 0 (0.00) | 0 (0.00) | 1 (100) | 1 (100) |
| Zyrtec | 0 (0.00) | 1 (100) | 0 (0.00) | 0 (0.00) | 1 (100) | 1 (100) |
| Total users | 121 (78.57) | 19 (12.33) | 12 (7.79) | 1.2(1.22) | 133 (86.36) | 154 (100) |

Note: % refers to the rate of users relative to the total number of users across each of the four groups. Total users is the total number of users observed across all four groups. The total number of unique users does not equal the users displayed due to the presence of unique users who participated across multiple groups.

|  | Group 1 Users | Group 2 Users | Group 3 Users | Group4 Users | Unique Users | Total  Users |
| --- | --- | --- | --- | --- | --- | --- |
| *Diagnosis, n (%)* | | | | | | |
| Myasthenia Gravis | 56 (80.00) | 11 (15.71) | 3 (4.28) | 0 (0.00) | 61 (87.14) | 70 (100) |
| Covid | 6 (80.00) | 1 (10.00) | 1(10.00) | 0 (0.00) | 8 (100) | 8 (100) |
| Thymoma | 3 (60.00) | 1 (20.00) | 1 (20.00) | 0 (0.00) | 5 (100) | 5 (100) |
| Sleep Apnea | 3 (100) | 0 (0.00) | 0 (0.00) | 0 (0.00) | 3 (100) | 3 (100) |
| Lambert-Eaton syndrome | 3 (100) | 0 (0.00) | 0 (0.00) | 0 (0.00) | 3 (100) | 3 (100) |
| Amyotrophic lateral sclerosis | 1 (50.00) | 1 (50.00) | 0 (0.00) | 0 (0.00) | 2 (100) | 2 (100) |
| Viral Infection | 2 (100) | 0 (0.00) | 0 (0.00) | 0 (0.00) | 2 (100) | 2 (100) |
| Sepsis | 1 (100) | 0 (0.00) | 0 (0.00) | 0 (0.00) | 1 (100) | 1 (100) |
| Anemia | 1 (100) | 0 (0.00) | 0 (0.00) | 0 (0.00) | 1 (100) | 1 (100) |
| Influenza | 2 (100) | 0 (0.00) | 0 (0.00) | 0 (0.00) | 2 (100) | 2 (100) |
| Cricopharyngeal Spasm | 1 (50.00) | 1 (50.00) | 0 (0.00) | 0 (0.00) | 2 (100) | 2 (100) |
| Lupus | 2 (100) | 0 (0.00) | 0 (0.00) | 0 (0.00) | 2 (100) | 2 (100) |
| Multiple Myeloma | 1 (100) | 0 (0.00) | 0 (0.00) | 0 (0.00) | 1 (100) | 1 (100) |
| Stiff Person Syndrome | 0 (0.00) | 0 (0.00) | 1 (100) | 0 (0.00) | 1 (100) | 1 (100) |
| Scleritis | 1 (100) | 0 (0.00) | 0 (0.00) | 0 (0.00) | 1 (100) | 1 (100) |
| Ehlers-Danlos | 1 (100) | 0 (0.00) | 0 (0.00) | 0 (0.00) | 1 (100) | 1 (100) |
| Diabetes | 1 (100) | 0 (0.00) | 0 (0.00) | 0 (0.00) | 1 (100) | 1 (100) |
| Erythromelalgia | 1 (100) | 0 (0.00) | 0 (0.00) | 0 (0.00) | 1 (100) | 1 (100) |
| Paraneoplastic Syndrome | 1 (100) | 0 (0.00) | 0 (0.00) | 0 (0.00) | 1 (100) | 1 (100) |
| Hiatal Hernia | 0 (0.00) | 1 (100) | 0 (0.00) | 0 (0.00) | 1 (100) | 1 (100) |
| Sinus Infection | 1 (100) | 0 (0.00) | 0 (0.00) | 0 (0.00) | 1 (100) | 1 (100) |
| Hyperparathyroidism | 1 (100) | 0 (0.00) | 0 (0.00) | 0 (0.00) | 1 (100) | 1 (100) |
| Tolosa-Hunt Syndrome | 1 (100) | 0 (0.00) | 0 (0.00) | 0 (0.00) | 1 (100) | 1 (100) |
| Asthma | 1 (100) | 0 (0.00) | 0 (0.00) | 0 (0.00) | 1 (100) | 1 (100) |
| Multiple Sclerosis | 1 (100) | 0 (0.00) | 0 (0.00) | 0 (0.00) | 1 (100) | 1 (100) |
| Bacterial infection | 1 (100) | 0 (0.00) | 0 (0.00) | 0 (0.00) | 1 (100) | 1 (100) |
| Oral Lichen Planus | 1 (100) | 0 (0.00) | 0 (0.00) | 0 (0.00) | 1 (100) | 1 (100) |
| Botulism Poisoning | 1 (100) | 0 (0.00) | 0 (0.00) | 0 (0.00) | 1 (100) | 1 (100) |

## **Supplement 9.** Diagnosis questions frequencies

**Table 18.** - Frequency of Users posting diagnosis questions

## **Supplement 9.** Diagnosis questions frequencies (continued)

|  | *Group 1 Users* | *Group 2 Users* | *Group 3 Users* | *Group 4 Users* | *Unique Users* | *Total*  *Users* |
| --- | --- | --- | --- | --- | --- | --- |
| *Diagnosis, n (%)* | | | | | | |
| Parkinsons | 1 (100) | 0 (0.00) | 0 (0.00) | 0 (0.00) | 1 (100) | 1 (100) |
| Vitiligo | 1 (100) | 0 (0.00) | 0 (0.00) | 0 (0.00) | 1 (100) | 1 (100) |
| Chronic obstructive pulmonary disease | 1 (100) | 0 (0.00) | 0 (0.00) | 0 (0.00) | 1 (100) | 1 (100) |
| Neuromyotonia Isaac's syndrome | 1 (100) | 0 (0.00) | 0 (0.00) | 0 (0.00) | 1 (100) | 1 (100) |
| Mitochondria | 1 (100) | 0 (0.00) | 0 (0.00) | 0 (0.00) | 1 (100) | 1 (100) |
| Intracranial Hypertension | 1 (100) | 0 (0.00) | 0 (0.00) | 0 (0.00) | 1 (100) | 1 (100) |
| Acute nasopharyngitis (common cold) | 1 (100) | 0 (0.00) | 0 (0.00) | 0 (0.00) | 1 (100) | 1 (100) |
| Mild Illness (unspecified) | 1 (100) | 0 (0.00) | 0 (0.00) | 0 (0.00) | 1 (100) | 1 (100) |
| Ulcerative Colitis | 1 (100) | 0 (0.00) | 0 (0.00) | 0 (0.00) | 1 (100) | 1 (100) |
| Miochonrdrial Encephalopathy | 1 (100) | 0 (0.00) | 0 (0.00) | 0 (0.00) | 1 (100) | 1 (100) |
| Mono | 1 (100) | 0 (0.00) | 0 (0.00) | 0 (0.00) | 1 (100) | 1 (100) |
| Whiplash | 1 (100) | 0 (0.00) | 0 (0.00) | 0 (0.00) | 1 (100) | 1 (100) |
| Lyme | 0 (0.00) | 0 (0.00) | 1 (100) | 0 (0.00) | 1 (100) | 1 (100) |
| Nephrotic Syndrome | 1 (100) | 0 (0.00) | 0 (0.00) | 0 (0.00) | 1 (100) | 1 (100) |
| Malaria | 1 (100) | 0 (0.00) | 0 (0.00) | 0 (0.00) | 1 (100) | 1 (100) |
| Arthritis | 1 (100) | 0 (0.00) | 0 (0.00) | 0 (0.00) | 1 (100) | 1 (100) |
| Total Users | 111 (82.83) | 15 (11.19) | 8 (5.97) | 0 (0.00) | 115  (85.82) | 134 (100) |

**Table 18.** - Frequency of Users posting diagnosis questions (continued)

Note: % refers to the rate of users relative to the total number of users across each of the four groups. Total users is the total number of users observed across all four groups. The total number of unique users does not equal the users displayed due to the presence of unique users who participated across multiple groups.

**Table 19.** Location question multiple group users

| *Category* | *Multiple Groups Users* | *Groups Used* |
| --- | --- | --- |
| Myasthenia Gravis | 1 User | User 1 – Groups 2,3 |
| Cricopharyngeal Spasm | 1 User | User 1 – Groups 1,2 |

Note: this table depicts users of multiple groups who asked the same type of question across multiple groups.
